# Do chronic disease patients value generic health states differently from individuals with no chronic disease? A case of a multicultural Asian population

**DOI:** 10.1186/s12955-014-0200-6

**Published:** 2015-01-23

**Authors:** Mihir Gandhi, Julian Thumboo, Nan Luo, Hwee-Lin Wee, Yin-Bun Cheung

**Affiliations:** Biostatistics, Singapore Clinical Research Institute, Singapore, Singapore; Centre for Quantitative Medicine, Duke-NUS Graduate Medical School, Singapore, Singapore; Department of International Health, School of Medicine, University of Tampere, Tampere, Finland; Department of Rheumatology and Immunology, Singapore General Hospital, Singapore, Singapore; Department of Medicine, Yong Loo Lin School of Medicine, National University of Singapore, Singapore, Singapore; School of Public Health, National University of Singapore, Singapore, Singapore; Department of Pharmacy, Faculty of Science, National University of Singapore, Singapore, Singapore

**Keywords:** Chronic disease, EQ-5D, Utility, Valuation

## Abstract

**Background:**

There is conflicting evidence as to whether patients with chronic disease value hypothetical health states differently from individuals who have not experienced any long-lasting diseases. Furthermore, most studies regarding this issue have been conducted in western countries, with only one conducted in Asia. We aimed to evaluate possible systematic differences in the valuation of EuroQol Group five dimensions 3-level (EQ-5D-3L) health states by chronic disease patients and a population with no chronic disease in Singapore.

**Methods:**

A face-to-face survey for the valuation of the 42 health states of the EQ-5D-3L using the visual analogue scale (VAS) method was conducted in Singapore. The survey also asked participants to report any chronic diseases they had. Ordinary least-square regression models were employed to assess possible differences in the valuation scores of all health states, severe health states and non-severe health states by individual chronic disease patient groups (diabetes, rheumatism, hypertension, heart diseases and lung diseases) and by a group of participants with no chronic disease. A difference of 4 to 8 points on the 100-point VAS was considered to be of practical significance.

**Results:**

The analysis included 332 participants with at least one chronic disease and 651 participants with no chronic disease. After taking health state descriptors and covariates into account, mean valuation scores of the 42 health states by the heart disease group were higher by 4.6 points (p-value = 0.032) compared to the no chronic disease group. Specifically, the heart disease group valued severe health states 5.4 points higher (p-value = 0.025) than the no chronic disease group. There was no practically significant difference in the mean valuation score of non-severe health states between the heart disease group and the no chronic disease group. No practically significant differences were found in the mean valuation score of all health states, severe health states and non-severe health states between any other chronic disease group and the no chronic disease group.

**Conclusions:**

In Singapore, heart disease patients valued EQ-5D-3L severe health states differently from individuals with no chronic disease. Other chronic disease groups did not value EQ-5D-3L health states differently from the no chronic disease group.

## Background

There is conflicting evidence as to whether patients with chronic disease value their own health states differently from individuals who have not experienced any such diseases [1,2]. Similar conflicting results have been reported for how patients with chronic disease and individuals with no such disease experience value hypothetical health states [1,2]. The difference in valuation of health states between the patients and individuals with no disease experience may arise because the patients might have adapted to their condition or because individuals with no disease experience overestimate the impact of disease or disability on quality of life [3]. Most studies that have evaluated differences in valuations by these two groups have been conducted in western countries; only one study has reported on an Asian population [1,4]. This is important as there is evidence of meaningful differences between populations of different countries regarding the valuation of health states [5,6]. In addition, most of the studies that have compared the valuation by chronic disease patients and that of by a no chronic disease population have compared the valuation of only selected disease-related health states, without covering a range of mild to severe states. Only a few studies have investigated the potential systemic difference between valuation by specific chronic disease patients and individuals with no experience of chronic disease regarding health states with a wide range of severity [7,8].

Differences in valuation between chronic disease patients and individual with no chronic disease may affect the outcomes of analyses of healthcare interventions. Cost-utility analysis (CUA) is a cost-effectiveness analysis in which the effect of health-care interventions is measured in terms of quality-adjusted life-years (QALYs) gained. QALYs are estimated as the time spent in a health state (quantity of life) multiplied by its utility (quality of life). QALYs are also an important outcome for monitoring health status in individual patients, measuring population health and measuring the impact of health-care intervention in clinical studies [9]. The question of whose utility (general-population-derived or patient-derived) should be used in clinical decision making and economic evaluations of health-care interventions has been debated in the literature [10,11]. The answer depends on the purpose for which the utility is used and context in which it is used. A general population-derived utility is desirable when the utility is needed to inform decisions that allocate societal resources, while a patient-derived utility may be more appropriate when making treatment decisions guided by patient preferences. The Panel on Cost-Effectiveness in Health and Medicine in the United States and the National Institute for Health and Care Excellence in England and Wales recommend that a general-population-derived utility for health states be used for cost-effectiveness analyses [12,13]. However, the latest systematic review revealed that less than one-third of published CUAs use a general-population-derived utility; the remainder used a patient-derived utility, a clinician- or expert-derived utility, or authors’ judgments [14]. Many investigators use a patient-derived utility because they believe that patients who have experienced the disease conditions can appraise their conditions more accurately than individuals who have not experienced such conditions [15]. On the other hand, CUAs using a general population-derived utility can help broader system-level decision making to prioritize health care funding in order to maximize the benefit for patients with different medical conditions- considering patients’ as well as non-patients’ perspectives [11]. This is a recommended approach when the health care is funded by the public/tax payers. However, if the health care costs are mostly paid by patients themselves, patient-derived utility should be considered. In Singapore, more than 60% of the health care costs are borne by patients [16]; and therefore patient-derived utility is relevant.

Utilities of health states from generic quality of life instruments, such as the EuroQoL Group five dimensions (EQ-5D) or Short Form six dimension (SF-6D), are preferred over health states from disease-specific quality of life instrument for CUAs. Utilities of generic health states allow comparisons of the effects on quality of life of different health-care interventions in different diseases. Currently, the EQ-5D is the most commonly used generic instrument for CUAs [14].

The present study draws on data from a valuation study of EQ-5D 3-level (EQ-5D-3L) health states in the Singapore general population which involves self-reporting of chronic diseases. We aimed to explore whether there are systematic differences in values for health states elicited by specific chronic disease patients (CDP) and by the no chronic disease population (NCDP). We also explored how the most severe health state and unconscious state were valued in relation to dead state by specific CDP and NCDP.

## Methods

### Valuation survey procedures

In 2009, the EQ-5D-3L—using the visual analogue scale (VAS) method—was used to conduct a cross-sectional, face-to-face survey of health state valuation in a representative sample of 1034 participants from the general population of Singapore. Singapore is a multi-ethnic city state with a rapidly increasing aging population. Its population is 75% Chinese, 13% Malay, 9% Indian (mostly Tamil speaking) and 3% others [17]. A multi-stage sampling approach was used to randomly select residential blocks, within which households were selected. We interviewed potential participants (one per household) who satisfied the pre-set recruitment quotas for ethnicity (400 Chinese, 400 Malay, and 234 Indians), gender (50% Female) and age (30% of 21–34 years, 40% of 35–49 years, and 30% of 50+ years). Within each ethnicity, there was a quota that half of the participants would use English for the interview and the remaining half would use their native language (i.e., Mandarin for Chinese, Malay for Malays and Tamil for Indians).

The EQ-5D-3L consists of 5 dimensions (mobility, self-care, usual activities, pain/discomfort, and anxiety/depression) with 3 response levels for each dimension (1: no problems, 2: some problems, and 3: extreme problems). This instrument thus describes 243 health states. Each health state is represented by one response level from each of the 5 dimensions. For example, 11112 describes a health state with no problems on the first 4 dimensions and some problem related to anxiety/depression. A subset of 42 EQ-5D-3L states was selected based on the protocol of Dolan [18]. Immediate death and the most severe state (‘33333’) were labeled as ‘dead’ and ‘all-worst’, respectively. Each participant was asked to compare between ‘dying now’ and ‘living for the rest of his/her life in all-worst’, from which the less desirable state was assigned a value 0 on the VAS. Each participant was then asked to value a unique set of 6 states from the subset of EQ-5D-3L states and either ‘dead’ or the ‘all-worst’ state, whichever one was not valued earlier at 0. The unique set of 6 health states that was assigned to each participant included states that were spread widely over the valuation space. A 100-point “feeling thermometer” with endpoints of 100 (most desirable, i.e., perfect health) and 0 (least desirable) was used as a VAS. For the six assigned states, participants were required to indicate where they would rate each of the states on the “feeling thermometer” by imagining themselves in that state for the rest of their life without changing. The participants were allowed to value more than one health state at the same level of VAS. In addition to the six assigned states, ‘unconscious’ state was also valued.

Participants were also asked to report their chronic diseases. The list of chronic diseases included diabetes, high blood pressure (hypertension), heart diseases, stroke, asthma or other lung diseases, cancer, rheumatism/back pain or other bone or muscle illness, mental illness (e.g., depression, anxiety neurosis, schizophrenia) and other illness (e.g., kidney problems or dialysis).

This study was approved by the SingHealth Centralized Institutional Review Board.

### Analyses

The analysis included participants with diabetes, high blood pressure/hypertension, heart diseases, asthma/lung diseases, rheumatism/back pain/other bone-muscle illness, or no chronic disease. The number of participants with other chronic diseases was small (<10) and these participants were not included in the analyses described in this manuscript.

Participants who met the following criteria were excluded from our analysis: a) valued less than 3 health states, b) did not value ‘dead’ or ‘all-worst’ state, c) valued ‘dead’ or ‘all-worst’ or ‘unconscious’ state higher than all of the other states, d) gave the same valuation score to all the health states, e) self-reported or rated by the interviewers as having a poor understanding of the health states description or valuation tasks. The valuation score used in the analyses was ‘raw’ VAS valuation score, which ranged from 0 (worst possible score) to 100 (best possible score). There is no consensus among researchers or regulatory bodies regarding the optimal method of transforming the valuation scores into utility [19].

We performed a separate analysis to compare the valuations by participants in each of the CDP groups with those of the NCDP group. Each analysis included two ordinary least-square regression models. Model I was used for the comparison of overall difference in valuation scores (including all the health states) between each CDP group and the NCDP group. Model II was used for the comparison of the differences in valuation scores of non-severe health states and severe health states by including an interaction term between the indicator variable for severe health state (versus non-severe health state) and the indicator variable for the specific CDP group (versus the NCDP group). We considered health states with at least one dimension at level 3 as “severe” health states, and the remaining states as “non-severe”.

Model I was performed for the valuation score with an indicator variable representing a specific CDP group, the members of which might have co-morbid conditions, versus the NCDP group as the independent variable. The model adjusted for indicator variables that represented the level of severity in each dimension of the health states. That is, including 2 indicator variables for each of the 5 dimensions of EQ-5D-3L. Furthermore, we included an indicator variable (commonly called ‘N3’ in the cost-utility analysis literature) to take into account additional disutilities when a severe problem (level 3) was reported on at least one dimension [20]. Finally, the comparison adjusted for ethnicity, gender, age, marital status, education level, religion and house type because the CDP group being analyzed and NCDP group might differ in these background characteristics.

Model II further included an interaction term between the CDP group indicator and the N3 in Model I. In this model, the coefficient of the CDP group provides an estimate of the difference between valuation scores of *non-severe health states*; whereas its sum with the interaction term provides an estimate of the difference between valuation scores of *severe health states* by the specific CDP group and the NCDP group after taking the health state descriptors and participants’ background characteristics into account. Perfect health state, ‘unconscious’ state and ‘dead’ state were excluded from Models I and II. The perfect health state was assigned a valuation score of 100 for each participant. Since each of the participants valued 6 health states, we used the Eicker-Huber-White robust standard error for cluster data for statistical inference [21].

We compared the valuation of the ‘all-worst’ and ‘unconscious’ health states with the valuation of ‘dead’ state by each of the CDP groups and the NCDP group. The mean valuation scores of the ‘all-worst’ state and ‘unconscious’ state were compared with the ‘dead’ state using a paired t-test.

All the analyses were carried out using Stata/MP 10.1 for Windows. A minimally important difference of 4 to 8 points on the 100-point VAS was considered to be of practical significance [22-24].

## Results

All 1034 participants provided demographic and health characteristic information. Nine participants had chronic diseases other than diabetes, high blood pressure/hypertension, heart disease, asthma/lung disease or rheumatism/back pain/other bone-muscle illness. Thirty-four participants valued ‘dead’ state higher than all the other states, 3 participants valued ‘unconscious’ state higher than all the other states, 1 participant did not value the ‘all-worst’ state and 4 participants were observed to have a poor understanding of valuation tasks. Hence, a total of 51 participants were excluded from the analysis. Table 1 shows the demographic and health characteristics of the 983 participants that were included in the analysis. The percentage of participants who had good-to-excellent self-reported general health varied between 68% and 75% among the CDP groups (Diabetes: 70/102, Rheumatism: 122/162, Hypertension: 107/145, Heart diseases: 29/44, Lung diseases: 30/44) as compared to 95% among NCDP group (621/651). Participants in the CDP groups were older, had lower education level and lower self-reported general health compared to participants in the NCDP group.

**Table 1:** **Demographic and health characteristics of the study participants**

**Characteristics**	**All participants (N = 983)**	**No chronic disease group (N = 651)**	**Diabetes (N = 102)**		**Rheumatism (N = 162)**		**Hypertension (N = 145)**		**Heart diseases (N = 44)**		**Lung diseases (N = 44)**	
	**n (%)**	**n (%)**	**n (%)**	P-value*	**n (%)**	P-value*	**n (%)**	P-value*	**n (%)**	P-value*	**n (%)**	P-value*
Female	493 (50.2)	329 (50.5)	47 (46.1)	0.404	92 (56.8)	0.071	69 (47.6)	0.530	9 (20.5)	<0.001	29 (65.9)	0.044
Age (years)				<0.001		<0.001		<0.001		<0.001		0.048
21-29	190 (19.3)	168 (25.8)	0 (0.0)		7 (4.3)		2 (1.4)		0 (0.0)		14 (31.8)	
30-39	218 (22.2)	178 (27.3)	6 (5.9)		21 (13.0)		7 (4.8)		1 (2.3)		9 (20.5)	
40-49	261 (26.6)	181 (27.8)	22 (21.6)		43 (26.5)		24 (16.6)		7 (15.9)		5 (11.4)	
50-59	192 (19.5)	91 (14.0)	41 (40.2)		40 (24.7)		57 (39.3)		18 (40.9)		8 (18.2)	
60+	122 (12.4)	33 (5.1)	33 (32.4)		51 (31.5)		55 (37.9)		18 (40.9)		8 (18.2)	
Ethnicity				0.001		0.024		0.914		0.001		0.287
Chinese	363 (36.9)	234 (35.9)	26 (25.5)		67 (41.4)		56 (38.6)		9 (20.5)		16 (36.4)	
Malay	395 (40.2)	284 (43.6)	37 (36.3)		50 (30.9)		57 (39.3)		14 (31.8)		14 (31.8)	
Indian	225 (22.9)	133 (20.4)	39 (38.2)		45 (27.8)		32 (22.1)		21 (47.7)		14 (31.8)	
Education level				<0.001		<0.001		<0.001		<0.001		0.352
Primary (6 years) or less	187 (19.0)	74 (11.4)	48 (47.1)		60 (37.0)		58 (40.0)		22 (50.0)		12 (27.3)	
Secondary (11 years)	555 (56.5)	387 (59.5)	44 (43.1)		79 (48.8)		77 (53.1)		18 (40.9)		22 (50.0)	
Diploma/degree or higher	241 (24.5)	190 (29.2)	10 (9.8)		23 (14.2)		10 (6.9)		4 (9.1)		10 (22.7)	
Married/living with partner	739 (75.2)	481 (73.9)	84 (82.4)	0.090	128 (79.0)	0.233	120 (82.8)	0.022	38 (86.4)	0.106	23 (52.3)	0.001
Religion				0.051		0.094		0.796		0.017		0.894
Buddhism/Taoism	224 (22.8)	139 (21.4)	19 (18.6)		43 (26.5)		37 (25.5)		4 (9.1)		12 (27.3)	
Islam	410 (41.7)	289 (44.4)	41 (40.2)		53 (32.7)		63 (43.5)		17 (38.6)		16 (36.4)	
Hinduism/Sikhism	192 (19.5)	117 (18.0)	31 (30.4)		40 (24.7)		24 (16.6)		17 (38.6)		10 (22.7)	
Christianity	80 (8.1)	54 (8.3)	7 (6.9)		14 (8.6)		10 (6.9)		3 (6.8)		3 (6.8)	
No religion	77 (7.8)	52 (8.0)	4 (3.9)		12 (7.4)		11 (7.6)		3 (6.8)		3 (6.8)	
House type				0.377		0.175		0.786		0.204		0.533
Government owned: 4 rooms or smaller	668 (68.0)	449 (69.0)	71 (69.6)		106 (65.4)		96 (66.2)		25 (56.8)		27 (61.4)	
Government owned: 5 rooms or bigger	292 (29.7)	191 (29.3)	27 (26.5)		49 (30.3)		45 (31.0)		18 (40.9)		16 (36.4)	
Private	23 (2.3)	11 (1.7)	4 (3.9)		7 (4.3)		4 (2.8)		1 (2.3)		1 (2.3)	
General health status				<0.001		<0.001		<0.001		<0.001		<0.001
Excellent	97 (9.9)	85 (13.1)	3 (2.9)		5 (3.1)		2 (1.4)		2 (4.6)		2 (4.6)	
Very good	376 (38.3)	302 (46.4)	19 (18.6)		24 (14.8)		31 (21.4)		6 (13.6)		9 (20.5)	
Good	409 (41.6)	234 (35.9)	48 (47.1)		93 (57.4)		74 (51.0)		21 (47.7)		19 (43.2)	
Fair	93 (9.5)	30 (4.6)	27 (26.5)		36 (22.2)		32 (22.1)		13 (29.6)		11 (25.0)	
Poor	8 (0.8)	0 (0.0)	5 (4.9)		4 (2.5)		6 (4.1)		2 (4.6)		3 (6.8)	

*Comparison with the no chronic disease group using Fisher’s exact test.

Table 2 summarizes the comparison of health state valuation scores between the CDP groups and the NCDP group. Mean observed differences between the CDP groups and the NCDP group regarding the valuation score of all the 42 EQ-5D health states, non-severe health states and severe health states ranged from −3.3 to 0.5, −3.7 to −1.0 and −2.8 to 2.1, respectively. After taking health state descriptors and covariates into account in the regression analysis, the mean differences between the CDP groups and the NCDP group regarding valuation scores of all the health states ranged from −2.5 to 1.6 (each p-value >0.05), except for the heart disease group. The adjusted mean valuation score of all the health states for the heart disease group was 4.6 points higher (95% CI: 0.4 to 8.9; p-value = 0.032) than that of the NCDP group. Similarly, after taking health state descriptors and covariates into account, the mean differences between the CDP group and the NCDP group regarding severe health state valuation scores ranged from −2.4 to 1.8 (each p-value >0.05), except for the heart disease group. The adjusted mean valuation score of severe states for the heart disease group was 5.4 points higher (95% CI: 0.7 to 10.1; p-value = 0.025) than that of the NCDP group. After taking health state descriptors and covariates into account, there was no practically significant difference in the mean valuation scores of non-severe health states between any CDP group and the NCDP group. The changes in the mean differences after the adjustment for the covariates could be due to differences in the distribution of demographic characteristics between the CDP and NCDP groups (please see Table 1). For example, the NCDP group had more participants married/living with partners, which was associated with higher value, compared with unmarried participants in multivariable analysis. After statistical adjustment, the difference between NCDP and lung disease groups would become smaller. Similarly, the NCDP group had differences in multiple demographic characteristics, such as more female participants, fewer Indian participants, and more participants following Buddhism/Taoism, which were associated with higher value, compared to the heart disease group. Thus, after statistical adjustment, the heart disease group had higher valuation score compared to the NCDP group. Other demographic characteristics did not have much influence on the valuation score (Details not shown).

**Table 2:** **Comparison of valuation of health states between the chronic disease groups and the no chronic disease group taking into account health state descriptors and covariates**

**Health States**	**Diabetes (n = 102)**	**Rheumatism (n = 162)**	**Hypertension (n = 145)**	**Heart diseases (n = 44)**	**Lung diseases (n = 44)**	**No chronic disease (n = 651)**
All health states^1^						
Mean (SD)	43.6 (30.0)	43.4 (30.6)	44.4 (30.0)	43.1 (28.7)	40.6 (29.5)	43.9 (30.6)
Mean difference (95% CI)^2,3^	1.6 (−1.2, 4.3)	0.4 (−2.0, 2.8)	0.7 (−1.7, 3.1)	4.6 (0.4, 8.9)*	−2.5 (−6.2, 1.2)	-
Non-severe health states^1,4^						
Mean (SD)	71.6 (18.8)	71.3 (20.0)	71.2 (19.7)	69.8 (16.8)	69.0 (22.1)	72.7 (19.3)
Mean difference (95% CI)^2,3^	1.0 (−2.5, 4.6)	−0.3 (−3.4, 2.9)	−1.0 (−4.3, 2.3)	2.6 (−2.4, 7.6)	−2.6 (−9.0, 3.8)	-
Severe health states^1,4^						
Mean (SD)	31.4 (25.4)	31.9 (26.6)	33.6 (26.5)	33.7 (26.0)	28.7 (23.6)	31.5 (25.8)
Mean difference (95% CI)^2,3^	1.8 (−1.2, 4.7)	0.7 (−1.9, 3.3)	1.3 (−1.2, 3.9)	5.4 (0.7, 10.1)*	−2.4 (−6.1, 1.2)	-

^1^The study included 42 EQ-5D-3L health states, not including perfect health, unconscious and dead states. The perfect health state of EQ-5D-3L was assigned default value of 100 points on the visual analogue scale.

^2^Difference: mean scores of participants with chronic diseases minus mean scores of participants with no chronic disease.

^3^Using ordinary least square regression model adjusted for health state descriptors, disutility due to severe problems, ethnicity, gender, age, marital status, education level, religion and house type (see Methods section).

^4^EQ-5D-3L health states with at least one domain at severity level 3 are considered as ‘severe’ health states. Remaining health states are considered as ‘non-severe’ health states.

*P-value <0.05.

Table 3 summarizes the comparison of valuation scores for the ‘all-worst’ state with those of the ‘dead’ state by disease group. Except for the heart disease group, the mean difference in valuation scores of ‘all-worst’ state and ‘dead’ state by the CDP groups and the NCDP group were within the range of −8.4 points (95% CI: −11.8 to −4.9; p-value < 0.001) to −5.3 points (95% CI: −8.8 to −1.8; p-value = 0.003). For the heart disease group, this difference was −2.3 points (95% CI: −7.3 to 2.8; p-value = 0.370).

**Table 3:** **Comparison of valuation scores between the ‘all-worst’ state and the ‘dead’ state by disease group**

**Valuation Scores**	**Diabetes (n = 102)**	**Rheumatism (n = 162)**	**Hypertension (n = 145)**	**Heart diseases (n = 44)**	**Lung diseases (n = 44)**	**No chronic disease (n = 651)**
All-worst^#^						
Mean (SD)	3.3 (8.4)	2.1 (6.0)	3.1 (8.6)	4.8 (9.9)	1.7 (5.2)	3.1 (8.3)
Dead						
Mean (SD)	8.6 (13.3)	10.3 (15.9)	11.4 (17.1)	7.1 (10.4)	8.5 (10.9)	9.4 (14.1)
All-worst - Dead						
Mean difference (95% CI)	−5.3 (−8.8, −1.8)*	−8.1 (−11.0, −5.3)*	−8.4 (−11.8, −4.9)*	−2.3 (−7.3, 2.8)	−6.8 (−10.8, −2.7)*	−6.3 (−7.6, −4.9)*

^#^EQ-5L-3L health state with all its domains at severity level 3 is labelled as ‘all-worst’ health state.

*P-value < 0.05.

Table 4 summarizes the comparison of valuation scores for ‘unconscious’ state with those of the ‘dead’ state by disease group. Except for the heart disease group, the mean difference in valuation scores of ‘unconscious’ state and ‘dead’ state by the CDP groups and the NCDP group were within the range of −0.1 points (95% CI: −3.0 to 2.7; p-value = 0.922) to 3.0 points (95%CI: −0.1 to 6.0; p-value = 0.057). For the heart disease group, this difference was 4.2 points (95% CI: 0.4 to 8.0; p-value = 0.030).

**Table 4:** **Comparison of valuation scores between the ‘unconscious’ state and the ‘dead’ state by disease group**

**Valuation Scores**	**Diabetes (n = 102)**	**Rheumatism (n = 167)**	**Hypertension (n = 147)**	**Heart diseases (n = 46)**	**Lung diseases (n = 44)**	**No chronic disease (n = 667)**
Unconscious						
Mean (SD)	11.6 (11.1)	10.1 (12.3)	12.0 (13.0)	11.3 (11.7)	10.6 (9.6)	11.8 (12.6)
Dead						
Mean (SD)	8.6 (13.3)	10.3 (15.9)	11.4 (17.1)	7.1 (10.4)	8.5 (10.9)	9.4 (14.1)
Unconscious - Dead						
Mean difference (95% CI)	3.0 (−0.1, 6.0)	−0.1 (−3.0, 2.7)	0.6 (−2.6, 3.8)	4.2 (0.4, 8.0)*	2.1 (−1.4, 5.7)	2.4 (1.2, 3.6)*

*P-value < 0.05.

## Discussion

We examined the potential effect of experience with chronic disease on the valuation of EQ-5D-3L health states using the VAS method in a multicultural Asian population. Valuation by participants with five different types of chronic disease (diabetes, rheumatism, hypertension, heart disease and lung disease) was compared with valuation by participants with no chronic disease.

The heart disease group valued the health states 5 points higher than did the NCDP group (p-value = 0.032), which is mainly attributed to the heart participants’ valuation of the severe health states. This difference was statistically significant and larger than the minimal important difference of 4 points for the EQ-5D-3L valuation score, which indicates that the result is practically meaningful. The mean differences between the valuation by other CDP groups (diabetes, rheumatism, hypertension and lung diseases) and the NCDP group were all smaller than the minimal important difference.

The ‘all-worst’ state (the most severe state of EQ-5D with all dimensions at extreme severity) was valued worse than the ‘dead’ state by the majority of participants across the different types of chronic diseases and the NCDP group. Except for the heart disease group, all CDP groups and the NCDP group valued the ‘all worst’ state statistically and practically significantly lower than the ‘dead’ state. The heart disease group’s valuation of the ‘all-worst’ state was not statistically and practically significantly different from their valuation of the ‘dead’ state (difference = −2.3, p-value = 0.370).

We also found that the mean valuation score of the ‘unconscious’ state was likely to be equivalent to the ‘dead’ state by the NCDP group and all of the CDP groups except for the heart disease group. The difference in the mean valuation score of the ‘unconscious’ state and the ‘dead’ state by heart disease group was statistically significant and higher than the minimal important difference (difference = 4.2, p-value = 0.030), whereas the difference was statistically non-significant and less than 4 (minimal important difference) for the diabetes, rheumatism, hypertension, asthma/lung disease groups and the NCDP group.

A possible reason for heart disease patients giving higher valuation scores could be that a higher proportion of heart disease patients might have experienced one or more severe heath states, and this might have changed their perception regarding these health states. This might not be the case with other CDP groups and the NCDP group. On the other hand, the majority of CDP groups and the NCDP group might have experienced the non-severe health states, thus leading to their similar valuation of non-severe health states.

Wang et al. [5] in Singapore found that after adjusting for health state descriptors and demographic characteristics, there was no meaningful difference in the valuation of severe health states by diabetes patients and a population without diabetes. However, the study reported that diabetes patients valued the non-severe health states 13 points higher than did the no-diabetes population. Our findings do not fully support their results. It should be noted that Wang et al. included only 3 non-severe health states; hence their findings have limited applicability. On the other hand, we used 14 non-severe health states, which represent more generalized findings.

Our study findings are consistent with those of Pickard et al. [8]. Using the time trade-off method, Pickard et al. found no meaningful difference in valuation scores between CDP (arthritis, diabetes, depression, hay fever, cancer) and NCDP, except for heart failure patients [8]. Pickard et al. found that after adjusting for covariates, patients with heart failure only, and patients with heart failure and at least one other chronic disease, gave valuation scores higher by 25 points (n = 6, p-value = 0.222) and 7 points (n = 129, p-value = 0.049), respectively, compared to NCDP.

A possible explanation for no practical differences in the mean valuation score between individuals with chronic diseases and individuals without any chronic diseases might be because in this exercise, individuals with and without chronic diseases are valuing many hypothetical health states that are unlikely to reflect the actual health state(s) that one has experienced. As such, it is probably not surprising that generally speaking, individuals with chronic disease might value them similarly to individuals with no chronic disease.

This study has several potential limitations. First, the chronic disease conditions were self-reported by the participants. We did not collect any further information to confirm the disease, the severity of the disease or the time spent with the disease. Hence, there could be a chance of misclassification regarding reported diseases. A Finnish study showed that the sensitivity and specificity of self-reported chronic diseases (diabetes, hypertension, coronary heart disease, asthma and rheumatoid arthritis) could range from 78% to 96% and 96% to 99%, respectively [25]. This indicates a relatively large possibility that patients with chronic diseases could be misclassified into the NCDP group, but a small possibility that those with no chronic disease could be misclassified into a chronic disease group. This should mean that the difference between CDP and NCDP might be under-estimated but not over-estimated. Furthermore, this is a secondary analysis of existing data. The limited information related to disease conditions does not allow us to investigate any concrete reasons for the differences or lack of differences between the CDP and NCDP. Second, although our study had a sizable CDP group, nearly 80% of the CDP group self-reported their health status as good to excellent. Hence, our study findings may not be generalized to patients at a severe or unstable stage of chronic disease. Third, our study included only five chronic diseases (with a relatively small number of participants) and only one life-threatening chronic disease (heart diseases). Thus, our study findings may not be assumed to generalize to other life-threatening chronic diseases. Fourth, we performed separate statistical tests for comparing valuation by each CDP group with valuation by the NCDP group without multiplicity adjustment. Furthermore, the sample size was not powered for this analysis. Thus, the statistically significant findings might be due to inflated Type I error and therefore require further confirmation. Nevertheless, our findings are based on a random sample of a chronic disease population from the Asian general population. It also included many health states with a wide range of severity. It also has potential to generalize the findings for non-life-threatening chronic disease patients. We encourage conducting a larger study that includes a greater variety of life-threatening chronic disease patients, as well as varying severity levels and the verification of disease conditions and severity.

## Conclusions

Our study findings suggest that heart disease patients value severe EQ-5D-3L health states differently than individuals who have no experience with chronic disease when analyzed using the VAS method in a Singaporean population. However, the experience of chronic diseases other than heart disease does not necessarily result in a higher or lower valuation across all the health states of EQ-5D-3L.

## References

[1] Peeters Y, Stiggelbout AM (2010). Health state valuations of patients and the general public analytically compared: a meta-analytical comparison of patient and population health state utilities. Value Health.

[2] Dolders MG, Zeegers MP, Groot W, Ament A (2006). A meta-analysis demonstrates no significant differences between patient and population preferences. J Clin Epidemiol.

[3] Bremner KE, Chong CA, Tomlinson G, Alibhai SM, Krahn MD (2007). A review and meta-analysis of prostate cancer utilities. Med Decis Making.

[4] Wang P, Tai ES, Thumboo J, Vrijhoef HJ, Luo N. Does Diabetes Have an Impact on Health-State Utility? A Study of Asians in Singapore. Patient 2014 [Epub ahead of print] 10.1007/s40271-014-0059-y 24756482

[5] Luo N, Wang P, Thumboo J, Lim YW, Vrijhoef HJ (2014). Valuation of EQ-5D-3L health states in Singapore: modeling of time trade-off values for 80 empirically observed health states. Pharmacoeconomics.

[6] Johnson JA, Luo N, Shaw JW, Kind P, Coons SJ (2005). Valuations of EQ-5D health states: are the United States and United Kingdom different?. Med Care.

[7] Krabbe PF, Tromp N, Ruers TJ, van Riel PL (2011). Are patients’ judgments of health status really different from the general population?. Health Qual Life Outcomes.

[8] Pickard AS, Tawk R, Shaw JW (2013). The effect of chronic conditions on stated preferences for health. Eur J Health Econ.

[9] Kind P, Lafata JE, Matuszewski K, Raisch D (2009). The use of QALYs in clinical and patient decision-making: issues and prospects. Value Health.

[10] McTaggart-Cowan H (2011). Elicitation of informed general population health state utility values: a review of the literature. Value Health.

[11] Stamuli E (2011). Health outcomes in economic evaluation: who should value health?. Br Med Bull.

[12] Weinstein MC, Siegel JE, Gold MR, Kamlet MS, Russell LB (1996). Recommendations of the panel on cost-effectiveness in health and medicine. JAMA.

[13] National Institute for Health and Clinical Excellence (NICE): Guide to the Methods of Technology Appraisal. NICE; 2013 [http://www.nice.org.uk/article/pmg9/resources/non-guidance-guide-to-the-methods-of-technology-appraisal-2013-pdf] 27905712

[14] Brauer CA, Rosen AB, Greenberg D, Neumann PJ (2006). Trends in the measurement of health utilities in published cost-utility analyses. Value Health.

[15] Russell LB, Gold MR, Siegel JE, Daniels N, Weinstein MC (1996). The role of cost-effectiveness analysis in health and medicine. Panel on Cost-Effectiveness in Health and Medicine. JAMA.

[16] World Health Organization. World Health Statistics 2014. WHO Press. [Internet] http://apps.who.int/iris/bitstream/10665/112738/1/9789240692671_eng.pdf. Accessed November 14, 2014

[17] Singapore Department of Statistics. Census of Population 2010. Statistical Release 1. Demographic characteristics, education, language and religion. Singapore: Department of Statistics 2011 [Internet]. http://www.singstat.gov.sg/docs/default-source/default-document-library/publications/publications_and_papers/cop2010/census_2010_release1/cop2010sr1.pdf. Accessed April 16, 2014

[18] Dolan P (1997). Modeling valuations for EuroQol health states. Med Care.

[19] Lamers LM (2007). The transformation of utilities for health states worse than death: consequences for the estimation of EQ-5D value sets. Med Care.

[20] Szende A, Oppe M, Devlin N (2007). EQ-5D value sets: inventory, comparative review and user guide (EuroQol group monographs, Vol. 2).

[21] Williams R (2000). A note on robust variance estimation for cluster-correlated data. Biometrics.

[22] Walters SJ, Brazier JE (2005). Comparison of the minimally important difference for two health state utility measures: EQ-5D and SF-6D. Qual Life Res.

[23] Pickard AS, Neary MP, Cella D (2007). Estimation of minimally important differences in EQ-5D utility and VAS scores in cancer. Health Qual Life Out.

[24] Luo N, Johnson JA, Coons SJ (2010). Using instrument-defined health state transitions to estimate minimally important differences for four preferencebased health-related quality of life instruments. Med Care.

[25] Oksanen T, Kivimäki M, Pentti J, Virtanen M, Klaukka T, Vahtera J (2010). Self-report as an indicator of incident disease. Ann Epidemiol.

